# Modeling non-Gaussian data analysis on determinants of underweight among under five children in rural Ethiopia: Ethiopian demographic and health survey 2016 evidences

**DOI:** 10.1371/journal.pone.0251239

**Published:** 2021-05-13

**Authors:** Sara Abera Bekele, Moges Zerihun Fetene

**Affiliations:** 1 Statistics Department, Under Natural Science College, Wollo University, Wollo, Ethiopia; 2 Statistics Department, Under Natural and Computational Science College, University of Gondar, Gondar, Ethiopia; University of Western Australia, AUSTRALIA

## Abstract

**Background:**

Childhood under-nutrition is a major global health problem. Although the rate of under-nutrition in Ethiopia has declined in the last decade, but it still remains being the major causes of morbidity and mortality of children under-five years. The problem is even worse in rural areas. The prevalence of underweight among rural children was 25% compared with 13% among urban children. To alleviate this problem, it is necessary to determine the magnitude and determinants of underweight. The study models non-Gaussian data analysis to identify risk factors associated with underweight among under-five children in rural Ethiopia.

**Methodology:**

The data source for this study was secondary data, which was retrieved from EDHS 2016 database. It was analyzed using two model families; one with marginal models (GEE and ALR) in which responses are modeled and marginalized overall other responses, and the other is random effects model (GLMM) which is useful when the interest of the analyst lies in the individual’s response profiles as well as to evaluate within and between regional variations of underweight.

**Result:**

From fitting non-Gaussian data analysis to identify risk factors associated with underweight among under five children in rural Ethiopia, the independent variable which have significant effect on underweight were:—Age of child, birth interval, mothers education, fathers education, wealth index, diarrhea in last two weeks, fever in last two weeks are significant and also father’s work status shows that difference in significance among the category.

**Conclusion:**

Child age, preceding birth interval, mother’s education, household’s wealth index, fever, diarrhea, father’s education and father’s work status were associated with child underweight. Furthermore, there is both within and between regional heterogeneity of underweight among children in rural Ethiopia. Therefore, rigorous community-based interventions (such as uplifting mother’s education by providing formal education and preventing infectious diseases that cause diarrhea and fever) should be developed and executed throughout the country to improve this grave situation of underweight prevalence in rural areas of Ethiopia.

## Introduction

Nutrition is the sum total of the processes involved in the intake and utilization of food substances by living organisms, including ingestion, digestion, absorption, transport and metabolism of nutrients found in food [1].

Malnutrition refers to deficiencies, excesses or imbalances in a person’s intake of energy and/or nutrients. The term malnutrition covers two broad groups of conditions. One is ‘under nutrition’ which includes stunting (low height for age), wasting (low weight for height), underweight (low weight for age) which may also reflect wasting and/or stunting and micro-nutrient deficiencies or insufficiencies (a lack of important vitamins and minerals). The other is overweight, obesity and diet-related non-communicable diseases (such as heart disease, stroke, diabetes and cancer) [2].

Under nutrition is a condition in which the body does not have enough of the right kind of food to meet its energy, macro-nutrient (proteins, carbohydrates and fats) and micro-nutrient (vitamins and minerals) needs. Children can still be undernourished even if they have enough food to meet their energy requirements if that food lacks essential micro-nutrients. Over nutrition is a condition where the body has too much food, especially fats and sugars [3].

Malnutrition is the major cause of illness and death among under-five children in Ethiopia. The rate of malnutrition among under-five children in the country is among the highest in the world and Sub-Saharan Africa. Moreover, malnutrition is the underlying cause of about three-fifth of child death in the country. According to the Central Statistical Agency (CSA) report of the 2016 Ethiopian demographic and health survey (EDHS), 38%, 24%, and 10% of under-five children were stunted, underweight, and wasted, respectively. The prevalence of stunting has decreased considerably from 51% in 2005 to 38% in 2016, an average decline of more than 1 percentage point per year. On the other hand, the prevalence of wasting changed little over the same time period, with a wasting rate of 10% at the time of the EDHS 2016, which was 12% in 2005. The prevalence of underweight has consistently decreased from 33% to 24% over the 11-year period. This demonstrates promising progress in reducing levels of under-nutrition over the past decade. However, the baseline levels of under-nutrition remain so high that the country still needs to continue substantial investment in nutrition. The problem is even worse in rural areas. For instance, the prevalence of underweight among rural children was 25% compared with only 13% among urban children. This shows the existence of a huge gap in level of underweight between rural and urban under-five children in Ethiopia [4].

Even though the problem of child malnutrition in Ethiopia has been sufficiently documented, the reasons behind it are still poorly understood. There is also inconsistency across studies regarding the determinant factors behind childhood under-weight. Moreover, previous studies on this area in Ethiopia were considered about modeling only the fixed effects of covariates without including the random effects and with no considering sampling structures of data. Most of the studies previously done are simply using only the ordinary logistic regression model. This study, therefore, had filled the gaps in understanding the status of children underweight by identifying determinant factors of underweight in rural Ethiopia and assessing the performance of different models using clustered data from EDHS 2016. The study is modeling non-Gaussian data analysis to identify risk factors associated with underweight among under-five children in rural Ethiopia evidence of EDHS 2016.

## Methodology

### Data source

The source of the data for the study was secondary data, which was retrieved from EDHS 2016 database. Ethiopian DHS is part of the worldwide MEASURE DHS project funded by the United States Agency for International Development (USAID). It was conducted by the agencies of the Federal Ministry of Health and the Central Statistical Agency (CSA) of Ethiopia.

### Response variable

To determine the level of underweight, the variable was expressed as a dichotomous variable category 0 if not underweight (>-2SD) and category 1 if underweight (<-2SD).

$$Y_{i}=\left\{ \begin{array}{l} \;0,notunderweight>-2SD(-2\;Z-score)from\;the\;median\;of\;reference\\ 1,underweight<-2SD(-2\;Z-score)from\;the\;median\;of\;reference \end{array}\right.$$

Where, Yi is the status of underweight of i^th^ children measured as a dichotomous variable.

### Methods of data analysis

In this study, the marginal models such as Generalized Estimating Equations (GEE), Alternating Logistic Regression (ALR) and random effects model such as Generalized Linear Mixed Model (GLMM) were used to fit to the data.

#### Generalized Estimating Equation (GEE)

Generalized estimating equations (GEE) models are a direct extension of basic quasi-likelihood theory from cross-sectional to repeated or otherwise correlated measurements. They estimate the parameters associated with the expected value of an individual’s vector of binary responses and phrase the working assumptions about the association between pairs of outcomes in terms of marginal correlation [5].

#### Alternating Logistic Regression (ALR)

Generalized estimating equation (GEE), allow estimation of first and second moment parameters in regression models for multivariable binary data. When association among the observation is important and is measured using marginal odds ratios, the computations required will prevent the applications in studies with large clusters. An alternative approach that overcomes the computational limitations encountered in many problems is proposed what is called alternative logistic regression [6].

#### Generalized Linear Mixed Model (GLMM)

Generalized linear model (GLM) extend ordinary regression by allowing non-normal responses and a link function of the mean. The generalized linear mixed model is a further extension that permits random effects as well as fixed effects in the linear predictor [7]. Antonio and Beirlant defined GLMM as extend of GLM by allowing for random or cluster-specific effects in the linear predictor. These models are useful when the interest of the analyst lies in the individual responses profile rather than the marginal mean.

### Non-Gaussian modeling

Statistical modeling provides away to connect the data with the statistics. Based on the statistical properties of the observed data, an appropriate model can be chosen that leads to a promising practical performance. The Gaussian distribution is the most popular and dominant probability distribution used in statistics. However, various data in real applications have bounded support or semi-bounded support. As the support of the Gaussian distribution is unbounded, such type of data is obviously non-Gaussian distributed.

Ignoring the dependency of the observations will overestimate the standard errors of the time dependent predictors, since we have not accounted for between subject variability. Therefore, the model that we have used is useful for improving this problem of our variables of interest. Because our response variable is categorical variable that have time dependent predictors.

### Ethics approval

The study was based on publicly available data from the EDHS. Ethical approval was the responsibility of the agency which collected the data.

## Result

### Summary of descriptive statistics

The detailed summary of descriptive statistics are available in the Table 1 of S1 File with its description.

### Analysis of Generalized Estimating Equations (GEE)

Before selecting the correct correlation structure, consider the model building strategy (variable selection). This was done by considering two different working correlation assumptions (exchangeable and independence). This describes in Table 2 of S1 File.

From the result presented in Table 2 of S1 File, the exchangeable working correlation assumption was found to be plausible since the two standard errors were closer each other. After decide to working correlation type we have fitted both model of generalized estimating Equations and alternating logistic regression (ALR) by using the parsimonious correlation type. Analysis of GEE parameter estimates are available in Table 3 of S1 File.

### Analysis of Alternating Logistic Regression model (ALR)

#### Parameter interpretation of ALR model

The result for ALR analysis presented in Table 4 of S1 File reveals that, age is significantly related to underweight of children. This implies that after adjusting all other predictor variables in the model, the estimated odds of being underweight of under-five children in rural Ethiopia who had the age group 12 to 24 months was exp (0.831) = 2.296 (95% CI: 1.879, 2.801) times higher, the odds for age group of 24 to 36 months was exp(0.933) = 2.542 (95% CI: 2.094, 3.083) times higher, the odds for age group of 36 to 48 months was exp (0.813) = 2.255 (95% CI: 1.861,2.735) times higher and odds ratio for age group 48 to 59 months was exp (0.944) = 2.570 (95% CI: 2.102,3.142) times higher compared to the reference age category 0 to 12 months. This indicates that the probability of being underweight is approximately two folds more likely than reference age group.

From the result the researchers also observed that birth interval has statistically significant effect on the chance of being underweight for under-five children. Compared to children whose preceding birth interval was less than 33 months (the reference category), the odds of being underweight among children whose preceding birth interval was more than 33 months was exp (-0.305) = 0.737 (95% CI 0.653, 0.832) times lower. This indicates that the probability of being underweight among children whose preceding birth interval was more than 33 months is about 26% less as compared to children whose preceding birth interval was less than 33 months controlling for all the other variables in the model.

Statistically significant difference has been observed between father’s education level and underweight status of children. The odds of being underweight among children whose fathers had primary education level was exp(-0.229) = 0.795, (95% CI: 0.686, 0.923) times lower compared to children whose fathers had no formal education. This implies that the probability of being underweight is reduced by about 21% for children whose fathers had primary educational level when compared to children whose fathers had no formal education (the reference category) controlling for all the other variables in the model. There is no significant difference in the probability of being underweight between children whose fathers had no formal education and children whose fathers had secondary and above level of education.

There is also significant association between mother’s educational level and underweight status of children. The odds of being underweight among children whose mothers had primary education level was exp (-0.204) = 0.815, (95% CI: 0.679, 0.979) times lower compared to children whose mothers had no formal education. Likewise, the estimated odds of being underweight among children whose mothers had secondary and above education level was exp (-0.529) = 0.589, (95% CI: 0.356, 0.973), times lower than those children whose mothers had no formal education. This implies that the risk of being underweight among children whose mothers had primary education level and secondary and above education decreased by 18.5% and 41.1%, respectively, compared to children whose mothers had no formal education (the reference category) controlling for all the other variables in the model.

Employment status of father has also statistically significant association with underweight of children who resides in rural part of Ethiopia. The estimated odds of being underweight among children whose fathers are unemployed was exp (0.307) = 1.359, (95% CI: 1.111, 1.662), times higher than those children whose fathers are employed. This implies that the chance of being underweight for those children whose fathers are unemployed is 1.3 times more likely than children with employed fathers (the reference category) controlling for all the other variables in the model.

Children from the highest wealth index households are less likely to be underweight than those from the poor wealth indexed households. The estimated odds of being underweight among children residing in households with medium and rich wealth index was exp (-0.253) = 0.776 (95% CI: 0.641, 0.939) and exp (-0.656) = 0.519 (95% CI: 0.901, 0.636), respectively, times lower than children residing in households with poor wealth index. This means that the risk of being underweight among children from medium and rich wealth index are reduced by 22.4% and 48.1%, respectively, when compared with children residing in households with poor wealth index (the reference category) controlling for all the other variables in the model.

The finding of this study also identified that children’s diarrhea experience in the two weeks prior to survey administration is strongly associated with being underweight. The estimated odds of being underweight for children who had diarrhea for last two weeks before the survey was exp (0.3435) = 1.409 (95% CI: 1.175, 1.692) times higher than children who have no diarrhea for last two weeks before the survey. This implies that the likelihood of developing underweight is 1.4 times higher among children who had diarrhea before the survey as compared to children who have not had diarrhea in last two weeks before the survey (the reference category) controlling for all the other variables in the model.

As compared to children who have not had fever two weeks before the survey, the estimated odds of being underweight among children who had fever in last two weeks before the survey was exp (0.215) = 1.239 (95% CI: 1.034, 1.484) times higher. This means that the chance of developing underweight among children who had fever two weeks before the survey is 1.2 times higher compared to children who have not had fever two weeks before the survey (the reference category) controlling for all the other variables in the model.

The ALR model also provides the estimated constant log odds ratios (alpha). These measures provide information about the association between individual observations within the same cluster. This implies that, the estimated pair wise odds ratio relating two responses from the same cluster was exp (0.2345) = 1.264 (95% CI: 1.172, 1.363). These, the value of alpha which is greater than zero indicates that, the associations is found to be significant (p-value < .0001) and this means that there is a strong association between individual children about underweight in the same cluster.

### Analysis of Generalized Linear Mixed Model (GLMM)

#### Parameter interpretation of GLMM

The result for GLMM analysis presented in Table 6 of S1 File reveals that, given the same random effects b_j_, the estimated odds of being underweight of under five children in rural Ethiopia who had the age group 12 to 24 months was exp (0.862) = 2.368 (95% CI: 1.944, 2.886) times higher; the odds for age group of 24 to 36 months was exp (0.971) = 2.640 (95% CI: 2.175, 3.203) times higher, the odds for age group of 36 to 48 months was exp (0.845) = 2.328(95% CI: 1.915, 2.826) times higher and odds for age group 48 to 59 months was exp (0.988) = 2.686 (95% CI: 2.208, 3.271) times higher when compared with the reference age category 0 to 12 months in the same j^th^ cluster keeping constant the other fixed effect variables in the model. This implies that the probability of being underweight is approximately two folds more likely than reference age group 0 to 12 in the same cluster at the given random effects.

The result shows that birth interval has statistically significant effect on the chance of being underweight for under five children. Compared to children whose preceding birth intervals were less than 33 months (the reference category), the estimated odds of being underweight among children whose preceding birth intervals are more than 33 months was exp (-0.333) = 0.717 (95% CI: 0.632, 0.813) times lower in the same j^th^ cluster with constant random effect in the given cluster and the other fixed effect covariates in the model are constant. This indicates that the probability of being underweight among children whose preceding birth interval is more than 33 months was about 28% less as compared to children whose preceding birth intervals was less than 33 months in the same cluster at the given random effects.

Statistically significant difference has been observed between fathers’ education level and underweight status of children. At the given constant random effect, the odds of being underweight among children whose fathers had primary education level was exp(-0.204) = 0.815 (95% CI: 0.704, 0.944) times lower compared to children whose fathers had no formal education in the same cluster. This implies that the probability of being underweight is reduced by about 18% for children whose fathers had primary education level when compared with children whose fathers had no formal education (the reference category) in the same cluster with the same random effect. There is no significant difference in the probability of being underweight between children whose fathers had no formal education and children whose fathers had secondary and above level of education.

There is also significant association between mother’s education level and underweight status of children who live in rural Ethiopia. The odds of being underweight among children whose mothers had primary education level was exp(-0.210) = 0.810, (95% CI: 0.682, 0.963) times lower compared to children whose mothers had no formal education in the same cluster. Likewise, the estimated odds of being underweight among children whose mothers had secondary and above education level was exp (-0.506) = 0.603, (95% CI:0.363, 1.002), times lower than those children whose mothers had no formal education in the same cluster. This means that the risk of being underweight among children whose mothers had primary educational level and secondary and above education decreased by 19% and 40%, respectively, compared to children whose mothers had no formal education (the reference category) at the same cluster with the same random effect.

Employment status of fathers had also statistically significant association with underweight of children who are living in rural parts of Ethiopia. The estimated odds of being underweight among children whose fathers were unemployed was exp (0.261) = 1.298, (95% CI: 1.055, 1.597) times higher than those children whose fathers are employed (reference group) in the same cluster. This implies that the probability of being underweight for those children whose fathers were unemployed was 1.2 times more likely than children with employed fathers (the reference category) at the same cluster with the same random effect.

Another significant variable of children underweight was wealth index. The estimated odds of being underweight among children residing in households with medium and rich wealth index was exp (-0.267) = 0.766 (95% CI: 0.641, 0.415) and exp (-0.679) = 0.507 (95% CI: 0.417, 0.616), respectively, times lower than children residing in households with poor wealth index in the same cluster. This indicates that the risk of being underweight among children from medium and rich wealth index were reduced by 23.4% and 49.3%, respectively, when compared with children residing in households with poor wealth index (the reference category) in the same cluster at the given random effects.

The finding of this study also identified those children who had diarrhea two weeks before the survey was strongly associated with underweight. The estimated odds of being underweight for children who had diarrhea in last two weeks before the survey was exp (0.353) = 1.423 (95% CI: 1.173, 1.728) times higher than children who had no diarrhea in last two weeks before the survey in the same cluster. This implies that the likelihood of developing underweight is 1.4 times higher among children who had diarrhea before the survey as compared to children who have not had diarrhea in last two weeks before the survey (reference category) at the same cluster with the same random effect.

Similarly, as compared to children who had not fever two weeks before the survey, the estimated odds of being underweight among children who had fever in last two weeks before the survey was exp (0.239) = 1.269 (95% CI:1.057, 1.525) times higher. This implies that the chance of developing underweight among children who had fever two weeks before the survey was 1.2 times higher compared to children who had not fever two weeks before the survey (the reference category) in the same cluster at the given random effects.

The random effect parameters under GLMM were not estimable and thus it was not possible to interpret it. However, the estimates within and between standard deviation of random effects were 0.459 and 0.259 Which was larger than zero and the boundary of 95% confidence interval for the estimates does not contain zero. Therefore, it can be interpreted as there was significance heterogeneity or variation within and between regions on the underweight of children who are residing in rural part of Ethiopia.

The Q-Q plots for normality of random effects at regional and cluster levels are checked, thus, the fitted GLMM model is well fitted for the given data (see the Fig 1 in S1 File).

## Discussion

This study aims at modeling the underweight of children who live in rural parts of Ethiopia. As a preliminary analysis, assortments of summary statistics were employed to explore the proportion between the response variable of interest and available covariates. All the fitted models lead to the same conclusion that age of child, birth interval of child(in month), father’s educational status, mother’s educational status, father’s work status, wealth index of household, fever in last two weeks and diarrhea in last two weeks are significantly associated with underweight of under five children.

Age has a positive association with the underweight of under five children and children in higher age groups had higher odds of underweight. Which is consistent with the finding of [8–10]. This finding shows that later age within under 5 years’ age carry a higher risk of underweight. One possible explanation could be because of the late introduction of supplementary food with low nutritional quality. The other reason might be that a large portion of guardians in rural areas are neglecting to fulfill optimal food requirements of their children as the child’s age increases. Furthermore, when children start walking on their own they try to eat what they got in the field, thus increasing their exposure to infections and susceptibility to illness which will increase the risk of being underweight.

Moreover, birth interval of under five children had strong negative association with underweight. As the interval between children increases the possibility to be underweight decreases. This might be due to short birth interval between children might create sharing problems among living siblings and parents, can’t take better care of their children and compromise the breastfeeding duration of the index child. The mother herself may be biologically depleted from too frequent births, and this could also negatively affect the nutritional status of the newborn baby.

Parent education is a strong determinant of children nutritional status. That means higher educational status of parents is associated with better child background and care practices. Father’s educational status had significant negative association with underweight of under five children. This finding was consistent with reports from [11]. As father’s educational status increase the children probability to be underweight is decreased.

Similar with the previous studies [12, 13], this study confirms that a significantly negative association between education of mothers and the underweight of under five children. Education serves as vision for information and knowledge of available health care services and good feeding practices. Education also serves as substitute for women’s higher socioeconomic status that improves the ability of educated mothers to afford the cost of food items. Women’s education was found to be a strong determinant of present of underweight for under five children.

The other finding of this study is fathers work status or employment status also had strong positive association with underweight. The probability of being underweight for a child who had unemployed father is more than who have employed father.

In addition, this study reveals that under-five children from poor households are at a higher risk of underweight than children from rich households. This finding is consistent with other studies from Bangladesh and Ethiopia [8, 14, 15]. They indicated that better of households had better access to food and higher cash incomes than poor households, allowing them a quality diet, better access to medical care and more money to spend on essential non-food items such as schooling, clothing and hygiene products. Generally household wealth status is found to be associated with underweight, the more the household income, the less likely the child will become underweight.

As result showed from this study existence of diarrhea two weeks before the survey is significantly associated with underweight status in children under-five. This finding is consistent with other studies conducted in Ethiopia and Vietnam [12, 16]. The possible reason is that diarrhea plays a major role in the etiology of underweight because it results in increased needs and high energy expenditure, lower appetite and nutrient losses.

Finally the result of this study is vary from the existing study results. This study were includes variables which is not included in the previous study like birth interval and father’s work status. The result of this study is updated, because things are changed time to time.

## Conclusion

Two marginal models, GEE and ALR, have been compared for the analysis of marginal or population average effects of covariates on the response variable and it was obtained that, ALR model with measure of association exhibited the best fit for this data than GEE models. Moreover, Cluster specific (GLMM) model was applied for the purpose of including random effect parameters specific to clusters, which are directly used in modeling the random variation in the dependent variable at different levels of the data. The study revealed that GLMM, with two random intercept model was found to be appropriate for the analysis of within and between regional variations for underweight among under five children who lives in rural part of Ethiopia. This concludes that there is heterogeneity among children between regions as well as within regions.

The study identified that that age of child, birth interval, educational status of mother, educational status of fathers, work status of fathers, wealth index, diarrhea in the two weeks before survey and fever were statistically significant risk factors for underweight of children in rural Ethiopia. The study also shows that there is region-wise inequality in children’s health, in addition there is within and between regional heterogeneity of underweight among children in rural Ethiopia.

### Limitation of the study

There are other factors associated with under-weight as indicated by different studies in different countries, this study is undertaken to explore a few of factors in rural parts of Ethiopia. Since this study was based on secondary data from EDHS, 2016, we studied only the variables which are included in the questionnaire.

## Supporting information

S1 File (DOCX)
