# Supplementary material for: Modeling non-Gaussian data analysis on determinants of underweight among under five children in rural Ethiopia: Ethiopian demographic and health survey 2016 evidences
Source: PLoS One. 2021 May 13;16(5):e0251239. doi: 10.1371/journal.pone.0251239 (PMC8118542; doi:10.1371/journal.pone.0251239)
Supplement: S1 File — (DOCX) [file pone.0251239.s001.docx]

***Supporting Information for “Modeling Non-Gaussian Data Analysis on Determinants of Underweight Among Under Five Children in Rural Ethiopia: Ethiopian Demographic and Health Survey 2016 Evidences”***

#### Sara Abera Bekele^1^, Moges Zerihun Fetene ^2*^

#### ^1^Statistics Department, Under Natural Science College, in Wollo University, Wollo, Ethiopia.

^2^Statistics Department, Under Natural and Computational Science College, in University of Gondar, Gondar, Ethiopia.

*Corresponding Author: Email: [kidankikone@gmail.com](mailto:kidankikone@gmail.com)

Sara Abera Bekele: Email: [Saronkal27@gmail.com](mailto:Saronkal27@gmail.com)

### Summary of descriptive statistics

Table 1 presents basic descriptive information that summarizes the percentage difference between the determinant factors and underweight of under five children who lives in rural part of Ethiopia. The study includes 7273 children who lives in rural part of Ethiopia from nine regional states and one city administrations in Ethiopia. The highest proportion of underweight children was observed among those whose age group is 48-59 months (30.7%) as opposed to the smallest percentage (14.2%) of underweight children which was observed among those whose age group is less than one year. The proportion of underweight children among those whose age group is 12-24 months is about 25.8%. For the age categories 24-36 and 36-48 the percentage of underweight is 28.3% and 26% respectively.

The proportion of underweight children also varies between mother’s educational levels. The proportion of underweight child is highest (26.7%) for those whose mother had no formal education compared to child whose mother had primary (17.8%) and secondary and above (8.1%) educational level. Similarly, the percentage of underweight children for those whose fathers have no formal, primary and secondary education and above educational level are about 28%, 20.9% and 17.5%, respectively.

With regard to household wealth index, the highest proportion of the underweight children are from poor households (28.5%) whereas the lowest proportion of the underweight children (17.4%) are recorded from children residing in rich households.

Children who had diarrhea in last two weeks before the survey are more underweight (31.5%) than who did not have diarrhea (23.6%).The proportion of children who are underweight among those who had fever two weeks before the survey is (29.1%) and this is higher than for those who did not have fever two weeks before the survey (23.8%). About 27.5% of children who have <33 months birth interval are underweight, which is higher than the proportion of underweight children who are from >33 month birth interval (21.6%). The proportion of underweight children is almost the same (about 24%) for whose fathers are employed and unemployed.

Table 1: Summary of Descriptive Statistics

|  |  | **Underweight** | |  |
| --- | --- | --- | --- | --- |
| **Variables** | **Levels** | **Count** | **%** | **Total** |
| **Fwork status** | Unemployed | 145 | 24.3 | 597 |
|  | Employed | 1639 | 24.6 | 6676 |
| **Birth interval** | <33 month | 989 | 27.5 | 3591 |
|  | >33 month | 795 | 21.6 | 3682 |
| **Fever** | No | 1486 | 23.8 | 6250 |
|  | Yes | 298 | 29.1 | 1023 |
| **Age of child** | 0-12 | 243 | 14.2 | 1708 |
|  | 12-24 | 361 | 25.8 | 1398 |
|  | 24-36 | 374 | 28.3 | 1321 |
|  | 36-48 | 374 | 26.0 | 1441 |
|  | 48-59 | 432 | 30.7 | 1405 |
| **Wealth index** | Poor | 1085 | 28.5 | 3805 |
|  | Medium | 388 | 23.0 | 1684 |
|  | Rich | 311 | 17.4 | 1784 |
| **Meducation** | No education | 1503 | 26.7 | 5637 |
|  | Primary | 272 | 17.8 | 1525 |
|  | Sec. and above | 9 | 8.1 | 111 |
| **Feducation** | No education | 1106 | 28.0 | 3952 |
|  | Primary | 597 | 20.9 | 2858 |
|  | Sec. and above | 81 | 17.5 | 463 |
| **Diarrhea** | No | 1509 | 23.6 | 6400 |
|  | Yes | 275 | 31.5 | 873 |

Table 2: Empirical and model based standard errors for two proposed working correlations

| Exchangeable Independent | | | | | | |
| --- | --- | --- | --- | --- | --- | --- |
| Coefficient | Estimate | Model based S.E | Empirical  S.E | Estimate | Model  based S.E | Empirical S.E |
| β_0_ | -1.9126 | 0.1163 | 0.1262 | -1.8643 | 0.0967 | 0.1285 |
| β_1_ | 0.8312 | 0.0974 | 0.1019 | 0.8327 | 0.0974 | 0.1012 |
| β_2_ | 0.9359 | 0.0955 | 0.0987 | 0.9480 | 0.0600 | 0.0988 |
| β_3_ | 0.8154 | 0.0958 | 0.0985 | 0.8063 | 0.0693 | 0.1001 |
| β_4_ | 0.9442 | 0.0968 | 0.1028 | 0.9486 | 0.1193 | 0.1030 |
| β_5_ | 0.3054 | 0.0606 | 0.0620 | 0.2754 | 0.0832 | 0.0637 |
| β_6_ | -0.2301 | 0.0713 | 0.0758 | -0.2292 | 0.2503 | 0.0750 |
| β_7_ | -0.1116 | 0.1220 | 0.1330 | -0.1352 | 0.0966 | 0.1404 |
| β_8_ | -0.2064 | 0.0846 | 0.0932 | -0.2086 | 0.0882 | 0.0941 |
| β_9_ | -0.5211 | 0.2519 | 0.2550 | -0.5137 | 0.1093 | 0.2591 |
| β_10_ | -0.3075 | 0.0995 | 0.1029 | -0.3653 | 0.0889 | 0.1044 |
| β_11_ | 0.2562 | 0.0907 | 0.0974 | 0.2662 | 0.0676 | 0.0992 |
| β_12_ | -0.3980 | 0.1113 | 0.1107 | -0.4181 | 0.0947 | 0.1110 |
| β_13_ | 0.2132 | 0.0887 | 0.0916 | 0.1837 | 0.0967 | 0.0921 |
| β_14_ | 0.3438 | 0.0944 | 0.0929 | 0.3612 | 0.0600 | 0.0933 |

### Analysis of Generalized Estimating Equations (GEE)

Table 3: Analysis of GEE parameter estimates (empirical standard error estimates)

| Factor | Level | Parameter | Estimates  (s.e) | Log 95% conf.int | Z | Pr>\|Z\| |
| --- | --- | --- | --- | --- | --- | --- |
| Intercept |  | β_0_ | -1.913(0.116) | (-2.16,-1.66) | -15.2 | <.0001 |
| Age | 0-12(ref) |  |  |  |  |  |
|  | 12-24 | β_1_ | 0.831(0.102) | (0.632,1.031) | 8.16 | <.0001 |
|  | 24-36 | β_2_ | 0.936(0.099) | (0.743,1.129) | 9.49 | <.0001 |
|  | 36-48 | β_3_ | 0.815(0.098) | (0.622,1.008) | 8.28 | <.0001 |
|  | 48-59 | β_4_ | 0.944(0.103) | (0.743,1.144) | 9.18 | <.0001 |
| Birth interval | <33mont (ref) |  |  |  |  |  |
|  | >33month | β_5_ | -0.305(0.062) | (-0.427,0.184) | -4.93 | <.0001 |
| Feducation | Non educate(ref) |  |  |  |  |  |
|  | Primary | β_6_ | -0.230(0.076) | (-0.379,-0.081) | -3.03 | 0.0024 |
|  | Secondary and above | β_7_ | -0.112(0.133) | (-0.372,0.149) | -0.84 | 0.4016 |
| Meducation | Non educated(ref) |  |  |  |  |  |
|  | Primary | β_8_ | -0.206(0.093) | (-0.389,-0.024) | -2.21 | 0.0268 |
|  | Secondary and above | β_9_ | -0.521(0.255) | (-1.021,-0.021) | -2.04 | 0.0410 |
| Fwork status | Employed(ref) |  |  |  |  |  |
|  | Un employed | β_10_ | 0.307(0.103) | (0.106,0.508) | 2.99 | 0.0028 |
| Wealth | Poor(ref) |  |  |  |  |  |
|  | Medium | β_11_ | -0.256(0.097) | (0.447, 0.065) | -2.63 | 0.0085 |
|  | Rich | β_12_ | -0.654(0.104) | (-0.858,-0.450) | -6.29 | <.0001 |
| Fever | No(ref) |  |  |  |  |  |
|  | Yes | β_13_ | 0.213(0.092) | (0.034,0.393) | 2.33 | 0.0199 |
| Diarrhea | No(ref) |  |  |  |  |  |
|  | Yes | β_14_ | 0.344(0.929) | (0.162,0.525) | 3.70 | 0.0002 |
| QIC=7027.0858 | | | | | | |

Table 4: Analysis of ALR parameter estimates (empirical standard error estimates)

|  | Level | Parameter | Estimates  (s.e) | Log 95% conf.int | Z | Pr>\|Z\| |
| --- | --- | --- | --- | --- | --- | --- |
| Intercept |  | **β_0_** | -1.910(0.126) | (-2.158,-1.662) | -15.1 | <.0001 |
| Age | 0-12(ref) |  |  |  |  |  |
|  | 12-24 | **β_1_** | 0.831(0.101) | (0.631, 1.030) | 8.16 | <.0001 |
|  | 24-36 | **β_2_** | 0.933(0.099) | (0.739, 1.126) | 9.45 | <.0001 |
|  | 36-48 | **β_3_** | 0.813(0.098) | (0.621, 1.006) | 8.26 | <.0001 |
|  | 48-59 | **β_4_** | 0.944(0.103) | (0.743, 1.145) | 9.19 | <.0001 |
| Birth interval | <33month(ref) |  |  |  |  |  |
|  | >33month | **β_5_** | -0.305(0.062) | (-0.426,0.184) | -4.93 | <.0001 |
| Feducation | Non educate(ref) |  |  |  |  |  |
|  | Primary | **β_6_** | -0.229(0.076) | (-0.377,0.080) | -3.02 | 0.0025 |
|  | Secondary and above | **β_7_** | -0.114(0.133) | (-0.375,0.147) | -0.85 | 0.3931 |
| Meducation | Non educated(ref) |  |  |  |  |  |
|  | Primary | **β_8_** | -0.204(0.093) | (-0.387,0.021) | -2.19 | 0.0286 |
|  | Secondary and above | **β_9_** | -0.529(0.256) | (-1.032,0.027) | -2.07 | 0.0388 |
| Fworkstatus | Employed(ref) |  |  |  |  |  |
|  | Un employed | **β_10_** | 0.307(0.102) | (0.106,0.508) | 2.99 | 0.0028 |
| Wealth | Poor(ref) |  |  |  |  |  |
|  | Medium | **β_11_** | -0.253(0.097) | (-0.444, 0.062) | -2.60 | 0.0094 |
|  | Rich | **β_12_** | -0.656(0.104) | (-0.859, -0.452) | -6.32 | <.0001 |
| Fever | No(ref) |  |  |  |  |  |
|  | Yes | **β_13_** | 0.215(0.092) | (0.034, 0.395) | 2.34 | 0.0195 |
|  |  |  |  |  |  |  |
|  |  |  |  |  |  |  |
| Diarrhea | No(ref) |  |  |  |  |  |
|  | Yes | **β_14_** | 0.343(0.093) | (0.161, 0.526) | 3.69 | 0.0002 |
| Alpha |  | $\boldsymbol{\alpha}$ | 0.234(0.039) | (0.159,0.310) | 6.07 | <.0001 |
| QIC= 7027.1244 | | | | | | |

### Analysis of Alternating Logistic Regression Model (ALR)

Model building for ALR is follows the same procedure as the GEE model building strategy. First ALR model was fitted using all proposed covariates. Then the covariate with the large p-value is removed. Work status of mother, sex of child, source of drink water, birth order of child and number of house hold member are the removal covariates with Wald test (p-value > 0.05). The QIC values of both unreduced and reduced models are given by 7031.656 and 7027.124 respectively. Therefore, the reduced model with the eight covariates was considered as the best candidate model. Using the selected covariates and the association parameter α, alternating logistic regression (ALR) model that provides information about pair wise association of observations between two different individuals within the same cluster was fitted.

## Comparison of GEE and ALR Models

Since the likelihood function does not fully specified in marginal models, model comparison is based on quasi likelihood criteria (QIC) which is the modified AIC criteria. From the results presented in Table 3 and Table 4, we found that the QIC values are 7027.0858 and 7027.1244 for the GEE and ALR respectively. Since the two models have almost exactly the same QIC value both models are appropriate. However, the empirically corrected standard errors for ALR model are somewhat smaller than their counterpart under the GEE model. This implies that the ALR fits the data with small disturbance than GEE. Moreover, ALR extends beyond classical GEE in the sense that precision estimates follow for both the regression parameters β and the association parameters α. We are also in a position to emphasize that the association is strongly significant (p-value< 0.0001), provided it has been correctly specified, a declaration we could not make in the corresponding exchangeable GEE analysis.

Therefore, we can conclude that ALR is the better model for explaining the marginal association between underweight and the selected predictor variables. Thus, our interpretation of parameters is based on the ALR model.

## Analysis of Generalized Linear Mixed Model (GLMM)

### Model building in GLMM

In order to decide on the better of the two random effects models, two models were fitted, one the reduced model below with two random intercepts to estimate between and within regional variations and the other with one random intercept model to estimate within regional variation. AIC and Likelihood ratio test (LRT) were used to compared the two models in order to select an appropriate models.

Table 5: Information criteria for comparison of one and two random intercept models

| Models | AIC | BIC | -2loglik | Deviance | $\boldsymbol{\sigma}_{\mathbf{w}}$ | $\boldsymbol{\sigma}_{\mathbf{B}}$ | p-value |
| --- | --- | --- | --- | --- | --- | --- | --- |
| One int. | 6951.2 | 7065.9 | 3458.6 | 6917.2 | 0.5229 |  |  |
| Two int. | 6930.3 | 7051.8 | 3447.2 | 6894.3 | 0.4591 | 0.2594 | <0.0001 |

Where $\sigma_{w}$ and$\sigma_{B}$ are within and between regional variance respectively, and P is the p-value of the log likelihood ratio test of the two models. As we have seen from Table 5, the AIC of two random intercept model is reduced from 6951.2 to 6930.3, the BIC is reduced from 7065.9 to7051.8, the -2loglikelihood is reduced from 3458.6 to 3447.2 and the deviance of the model is reduced from 6917.2 to 6894.3. The small p-value of the log likelihood ratio test (p-value< 0.001) also indicates that the model with two random intercept is parsimonious model.

Also when considered a model without random effects (i.e simply the generalized linear model), it gives AIC value of 6931.6 which is large as compared to the above model with random effects. The resulting p-value (< 0001) of this test supports that considering the random effect model is essential. Therefore, we conclude that, the model with two random intercepts should be used to address the between and within-regional heterogeneity in the given data.

Next, the covariates for the fixed effect were assessed and the candidate covariates were selected by removing covariates starting from with highest p-value sequentially. Then the removable covariate from the first model is number of household member, birth order, source of drink water, work status of mothers, with the highest p-value and refitted the reduced model with the remaining covariates. The AIC is reduced from 6953.2 to 6930.3 and the p-value of log likelihood ratio test supports the reduced model is preferable one. The next removable variable is sex of child and toilet facility. Model with small number of covariates is considered to be preferable. Therefore, the model with covariates age of child, birth interval of child in month, father’s educational status, mother’s educational status, father’s work status, wealth index, diarrhea in last two weeks, fever in last two weeks before the survey are considered to be the most parsimonious model for GLMM. The parameter estimates and standard errors of the cluster-specific (GLMM) model are presented in Table 6.

Table 6: Parameter estimates (standard errors) and corresponding P value for GLMM

| Factor | Level | Parameter | Estimates(s.e) | Log95% conf.int | Z | Pr>\|Z\| |
| --- | --- | --- | --- | --- | --- | --- |
| Intercept |  | β_0_ | -1.670(0.156) | (-1.977,-1.364) | -10.68 | 0.002 |
| Ageofch | 0-12(ref) |  |  |  |  |  |
|  | 12-24 | β_1_ | 0.862(0.101) | (0.665,1.060) | 8.56 | 0.000 |
|  | 24-36 | β_2_ | 0.971(0.099) | (0.777,1.164) | 9.83 | 0.000 |
|  | 36-48 | β_3_ | 0.845(0.099) | (0.650,1.039) | 8.51 | 0.000 |
|  | 48-59 | β_4_ | 0.988(0.100) | (0.792,1.185) | 9.86 | 0.000 |
| Birth interval | <33month(ref) |  |  |  |  |  |
|  | >33month | β_5_ | -0.333(0.064) | (-0.458,-0.207) | -5.19 | 0.000 |
| Feducation | Non educate(ref) |  |  |  |  |  |
|  | Primary | β_6_ | -0.204(0.075) | (-0.351,-0.057) | -2.73 | 0.006 |
|  | Secondary and above | β_7_ | -0.059(0.129) | (-0.311,0.193) | -0.46 | 0.646 |
| Meducation | Non educate(ref) |  |  |  |  |  |
|  | Primary | β_8_ | -0.210(0.088) | (-0.382,-0.038) | -2.39 | 0.017 |
|  | Secondary & above | β_9_ | -0.506(0.259) | (-1.014,0.002) | -1.95 | 0.051 |
| Fwork status | Employed(ref) |  |  |  |  |  |
|  | Unemployed | β_10_ | 0.261(0.106) | (0.054,0.468) | 2.47 | 0.013 |
| Wealth | Poor(ref) |  |  |  |  |  |
|  | Medium | β_11_ | -0.267(0.091) | (-0.445,-0.88) | -2.93 | 0.003 |
|  | Rich | β_12_ | -0.679(0.099) | (-0.874,-0.485) | -6.85 | 0.001 |
| Fever | No(ref) |  |  |  |  |  |
|  | Yes | β_13_ | 0.239(0.093) | (0.056,0.422) | 2.56 | 0.010 |
| Diarrhea | No(ref) |  |  |  |  |  |
|  | Yes | β_14_ | 0.353(0.099) | (0.160,0.547) | 3.58 | <0.002 |
| $\boldsymbol{\sigma}_{\mathbf{B}}$ (Region) | Variance = 0.067 | Std.dev = 0.259 (0.146, 0.474) | | | | |
| $\boldsymbol{\sigma}_{\mathbf{w}}$ (Cluster) | Variance = 0.211 | Std.dev = 0.459 (0.363, 0.556) | | | | |

### Model Diagnostics

In GLMM, it is expected to assume that the random effects follow a normal distribution with mean zero and variance σ. Therefore, it is important to check whether this assumption is met or not. To check this assumption, the most commonly used method is aquantile-quantile (Q-Q) plot. When quantiles of two distributions are met, plotted dots face with the line *y = x*. A normal Q-Q plot is that which can be shaped by plotting quantiles of one distribution versus quantiles of normal distribution.  Thus,the fitted GLMM model is well fitted for the given data.


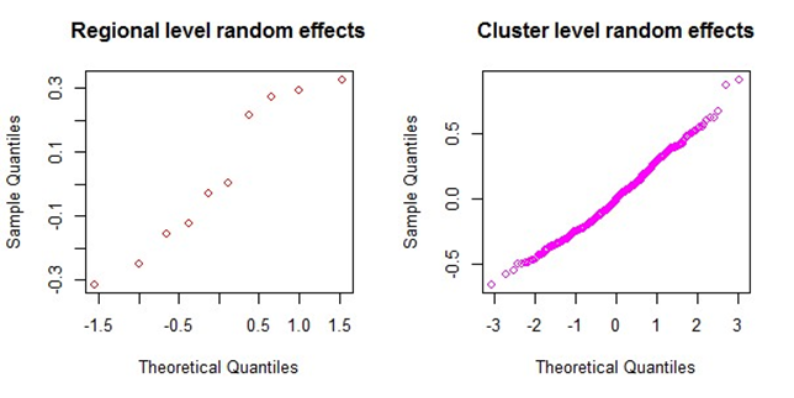


Figure 1: Diagnostic plots for the GLMM model
